# Whole-Genome Sequences of an Aerobic Anoxygenic Phototroph, *Blastomonas* sp. Strain AAP53, Isolated from a Freshwater Desert Lake in Inner Mongolia, China

**DOI:** 10.1128/genomeA.00071-13

**Published:** 2013-03-14

**Authors:** Yonghui Zeng, Michal Koblížek, Fuying Feng, Yapeng Liu, Zaohe Wu, Jichang Jian

**Affiliations:** a Guangdong Provincial Key Laboratory of Pathogenic Biology and Epidemiology for Aquatic Economic Animals, Guangdong Ocean University, Zhanjiang, China; b Institute of Microbiology CAS, Opatovický mlýn, Třeboň, Czech Republic; c Institute for Applied & Environmental Microbiology, Life Sciences College, Inner Mongolia Agricultural University, Huhhot, China

## Abstract

*Blastomonas* is a strictly aerobic bacteriochlorophyll *a*-producing genus within the alpha-4 *Proteobacteria*. Here we report the first genome sequence from this genus. The draft genome of *Blastomonas* sp. strain AAP53 contains a split photosynthesis gene cluster and two gene clusters encoding a flagellar system. Genes for the autotrophic CO_2_ fixation pathway are absent.

## GENOME ANNOUNCEMENT

The genus *Blastomonas* of the family *Sphingomonadaceae* (*Alphaproteobacteria*) is characterized by budding morphology and bacteriochlorophyll *a* synthesis under aerobic conditions. Only two type species have been reported in this genus, *B. natatoria* and *B. ursincola* (1–3). They are strictly aerobic chemoorganotrophs and facultative photoorganotrophs with motion ability by means of polar flagella. To assess the metabolic potentials and photosynthesis gene composition of *Blastomonas* species, we sequenced the whole genome of *Blastomonas* sp. strain AAP53. This yellow-pigmented isolate was collected in November 2011 from the surface water of the Swan Lake, a freshwater desert lake in the Inner Mongolia Autonomous Region, China.

*Blastomonas* sp. AAP53 was transferred five times on solid R2A media before inoculation into a liquid culture. DNA was extracted using a genomic DNA purification kit, and a 300-bp DNA library for Illumina sequencing technology was constructed. An Illumina HiSeq 2000 platform was employed for sequencing. Approximately 3.01 Gb raw data of 101-bp-long pair-end reads were generated. A total of 11,656,750 reads were subjected to quality control and trimming on the Galaxy server (4) and then were *de novo* assembled into contigs using the Velvet program (Ver. 1.2.08) (5). Various hash lengths between 31 and 91 were tested and an optimal assembly was achieved with a k-mer size of 57. Contigs less than 300 bp long were removed from the final assembly. Genome coverage was ca. 180×. Annotation was performed with the RAST (6) and BASys (7) servers.

The draft genome consisted of 28 contigs with an N_50_ value of 350,403 bp and a total length of 3.62 Mb, containing 3,369 open reading frames (ORFs) and 42 tRNAs. The G+C content was 63.6%. One complete rRNA operon was assembled. A full-length 16S rRNA gene sequence of this isolate shows 98.6% identity to that of *Blastomonas natatoria* strain DSM 3183. The photosynthesis gene cluster (PGC) was split and located on two separate contigs, probably due to insufficient sequencing coverage on the PGC region. A 13,758-bp-long contig contains the PGC genes of *bchIDO-crtF-bchCXYZ-pufBALMC*. Identified *pufBALM* protein sequences have 91 to 97% identity to those of *B. natatoria* and *B. ursincola*. A 55,061-bp-long contig contains the other part of PGC genes at its 3′ end, with a gene organization of *puhE*-acsF-ORF-*puhCBA-lhaA-bchMLHBNF*-ORF-*ppsR*-ORF-*bchG-pucC-bchP-tspO*-ORF.

Autotrophic CO_2_ fixation pathway genes are absent in this draft genome. There are genes encoding adenylyl-sulfate reductase, sulfite reductase, urea carboxylase, nitrite oxidoreductase, and nitrate reductase. Two gene clusters associated with the flagellar system were identified, with gene organizations of *flaA-fliA-flhA-flgJ*-ORF-*flgMA-*2×ORFs-*flgBCDEFGHIJKL-motAB* and *fliSD-flhB-fliRQPONMLK*-ORF-*fliIHGFE*. Various proteins that are involved in the aerobic respiratory electron transport chain were identified, including NADH dehydrogenase, cytochrome *c* oxidase, succinate dehydrogenase, and catalase, a finding that confirms the aerobic lifestyle of *Blastomonas* sp. AAP53. The presented genome sequences shall deepen our understanding of *Blastomonas* species and provide more insights into the metabolism and ecology of freshwater aerobic anoxygenic phototrophic (AAP) bacteria (8–11).

### Nucleotide sequence accession numbers.

This whole-genome shotgun project has been deposited at DDBJ/EMBL/GenBank under the accession number ANFZ00000000. The version described in this paper is the first version, ANFZ01000000.
